# Spatiotemporal Stability of Persistent Atrial Fibrillation Sources: Stable Source or Disease Progression?

**DOI:** 10.3390/jcdd13060256

**Published:** 2026-06-09

**Authors:** Rita B. Gagyi, Ioan A. Minciuna, Mate Vamos, Attila Nemes, Peter Ruppersberg, Wim Bories, Tamas Szili-Torok

**Affiliations:** 1 Department of Internal Medicine, Cardiology Centre, University of Szeged, Semmelweis Street 8, 6725 Szeged, Hungary; gagyi.rita@gmail.com (R.B.G.); 2 5th Department of Internal Medicine, Faculty of Medicine, “Iuliu Hațieganu” University of Medicine and Pharmacy, 400347 Cluj-Napoca, Romania; 3 Cortex Inc., Menlo Park, CA 94025, USA; 4 Acutus Medical Inc., 1930 Zaventem, Belgium

**Keywords:** persistent atrial fibrillation, electrographic flow, charge density, non-invasive mapping, panoramic atrial mapping

## Abstract

**Aims:** To assess the spatiotemporal stability of extra-pulmonary vein (PV) sources in patients with persistent atrial fibrillation (AF). **Methods and results:** Nine patients (mean age 63 ± 9 years, 55% male) with persistent AF were included who underwent an initial and at least one redo catheter ablation procedure utilizing panoramic atrial mapping (PAM) systems (CardioInsight, electrographic flow (EGF), and/or charge density (CDM) mapping). Procedures were performed in the following combinations: CDM-CDM (1 patient), CDM-EGF (1 patient), EGF-CDM (3 patients), CardioInsight-CDM (1 patient), EGF-EGF (3 patients). We reviewed maps and analyzed the location of AF sources. Spatiotemporal stability was defined as the presence of an AF source of identical location on available maps during the initial and the redo procedure. In 4 patients (44.4%), localization of AF sources mapped at the repeat procedure corresponded with the localization of sources mapped during the index procedure. In two patients, no sources were identified during the second procedure. In the remaining 3 patients, the localization of sources was detected at different locations. **Conclusions:** Our findings suggest the presence of spatiotemporal stability of AF sources; however, novel sources can also be found during the repeated procedure, consistent with disease progression.

## 1. Introduction

Catheter ablation (CA) for persistent atrial fibrillation (AF) is challenging and is associated with suboptimal long-term outcomes [1]. A major limitation of standard approaches to treat persistent AF is their predominantly empirical nature. Unlike for paroxysmal AF, pulmonary vein isolation (PVI) alone is considered insufficient for the majority of patients with persistent AF [2,3]. Adjunctive substrate ablation techniques to improve outcomes have been the subject of many clinical trials [4]. However, early results did not show major improvement in long-term outcomes compared with PVI-only procedures. Increasing evidence supports the existence of spatially localized extra-pulmonary-vein (PV) sources that maintain and initiate persistent AF [5]. Detailed characterization of these extra-PV sources remains sparse due to the limitations of currently used mapping technologies, which are unable to reproducibly detect and localize these sources. Hence, the spatiotemporal stability of AF maintaining extra-PV sources has never previously been characterized.

Electrographic flow mapping (EGF), charge density mapping (CDM), and CardioInsight are three novel, panoramic atrial mapping (PAM) based technologies specifically developed to identify AF sources [6]. Due to the unique technological features these mapping systems offer, both short-term and long-term outcomes of persistent AF ablation might improve. The aim of this case series was to assess the spatiotemporal stability of extra-PV sources mapped with PAM systems in patients with persistent AF.

## 2. Material and Methods

In this single-center case series, we included patients who underwent an initial and at least one redo CA for persistent AF using PAM. All procedures were performed under general anesthesia with the use of the robotic magnetic navigation (RMN) system between 2018 and 2023. Pre-operatively, the presence of intra-cardiac thrombus was evaluated by trans-esophageal echocardiography (TEE). When CardioInsight technology was utilized, a pre-procedural non-contrast chest CT scan was performed while a 252-electrode vest was placed on the patient’s torso. In this case, the mapping procedure took place on the same day as the CA procedure. The procedures typically started with (double) right femoral and (double) left femoral venous puncture to obtain venous access. Two 8 Fr sheaths were placed in the right femoral vein, one 6 Fr and one 10 Fr sheaths were inserted in the left femoral vein. After a decapolar diagnostic catheter was positioned in the coronary sinus (CS), an ICE-guided septal puncture was performed to obtain transseptal LA access using the NRG RF Transseptal needle (Baylis Medical, Mississauga, ON, Canada). A circular Lasso catheter (Biosense Webster, Diamond Bar, CA, USA) and a 4 mm tip Navistar RMT ThermoCool ablation catheter (Biosense Webster, Diamond Bar, CA, USA) were advanced into the left atrium via a SL-1 long sheath (St Jude Medical, St. Paul, MN, USA). Passive recrossing was performed with an Agilis 8.5 Fr medium curl sheath (Abbott, Chicago, IL, USA). PVI or PV re-isolation was performed with the following power settings: maximum radiofrequency energy application 45–55 W, with temperature limit 43 °C; and 17–30 mL/min irrigation.

After PVI was complete and confirmed to be intact with the entrance block, PAM was performed using one of the following systems: a 64-electrode basket catheter (FIRMap™; Abbott, Abbott Park, IL, USA) to perform EGF mapping, a 48-pole noncontact mapping catheter (AcQMap catheter, Acutus Medical, Carlsbad, CA, USA) to perform CDM and look for extra-PV sources of AF or the non-invasive CardioInsight mapping (described previously, Medtronic, Minneapolis, MN, USA). When using the basket catheters, we administered intravenous heparin for anticoagulation with a targeted ACT > 300 s. Activation maps were created during the procedure, and when an AF source was detected and localized, targeted ablation was performed with the above-mentioned power settings. In cases when EGF and CDM technologies were utilized, remapping and ablation were performed until no sources were seen or at the discretion of the operator using the same PAM system. Spatiotemporal stability was defined as the presence of an AF source of identical location on available maps during the initial and the redo procedure. ECV was performed when indicated. Sheaths and catheters were removed when electrical isolation of all PVs was complete at the end of the waiting time. All procedures were performed by the same team of highly experienced operators.

### 2.1. Panoramic Atrial Mapping Technologies

The CDM high-resolution non-contact mapping system (Acutus Medical, Carlsbad, USA) provides maps of electrical activation across an ultrasound-acquired cardiac chamber surface. The distal end of the diagnostic recording catheter (AcQMap catheter) is deployed into a 25 mm diameter spheroid, formed by six splines. Each spline has eight ultrasound transducers interspersed between eight biopotential electrodes, resulting in a total of 48 sensors of each type. The endocardial surface is reconstructed based on point-sets generated with the use of ultrasound imaging. The system samples up to 115,000 surface points per minute. From this ultrasound point set, the 3D surface is algorithmically reconstructed. The system acquires 150.000 intracardiac unipolar voltages/second to calculate cardiac activation as charge density via the inverse solution based on Poisson’s Equation. Activation maps are created within approximately 2 min and displayed as a spatiotemporal window of activation history across the reconstructed 3D anatomical image.

The EGF mapping system (Cortex, Menlo Park, CA, USA, version v9.0.2) records unipolar EGMs over 1 min using the above-described 64-pole basket mapping catheter. The software pre-processes unipolar EGMs to remove far-field artifacts and normalizes the signals to unitary amplitudes before they undergo flow analysis. Using Green’s algorithm, each 19 ms far-field corrected and normalized recording is summarized in a single minimal energy voltage map. Subsequent minimal energy voltage maps are then evaluated using a Horn-Schunck flow algorithm to determine whether the singularity (source) represents an active source or a passive rotational phenomenon. The EGF Summary map displays all active sources and passive rotations detected over the course of 1 min of recording.

The CardioInsight mapping system (Cleveland, OH, USA) is a noninvasive ECGI mapping system that uses body surface ECGs and a patient-specific anatomical model based on a CT scan input. The mapping procedure usually takes place on the same day as the ablation procedure. A 252-electrode vest is placed on the patient’s torso, and a high-resolution non-contrast computed tomography (CT) scan is performed to obtain the patient-specific cardiac anatomy and to define the position of each electrode on the torso. Atrial geometry is reconstructed in order to obtain a 3D mesh, which serves for the projection of unipolar signals. ECGs are acquired during the patient’s sinus rhythm and AF pre-procedurally or during the EP study. Activation maps are computed using intrinsic deflection-based methods from unipolar EGMs. The CardioInsight system uses reconstructed signals to calculate the phase of each signal. Cumulative maps are used to localize the areas of focal breakthrough and reentrant drivers.

Spatiotemporal stability was defined as the presence of an AF source localized within the same predefined anatomical atrial region or segment on the reconstructed chamber geometry during both the index and repeat procedures. Source comparison was therefore based on concordant regional anatomical localization rather than exact point-by-point spatial overlap.

### 2.2. Case Series

This case series describes nine patients undergoing an initial and a redo CA for persistent AF using the following PAM systems: CardioInsight, EGF, and AcQMap, in the following combinations: AcQMap-AcQMap (1 patient), AcQMap-EGF (1 patient), EGF-AcQMap (3 patients), CardioInsight-AcQMap (1 patient), EGF-EGF (3 patients). The mean age of the patients was 63 ± 9, and 55% of the patients were male. Demographic data and procedural properties for all patients are summarized in Table 1.

**Figure 1:**
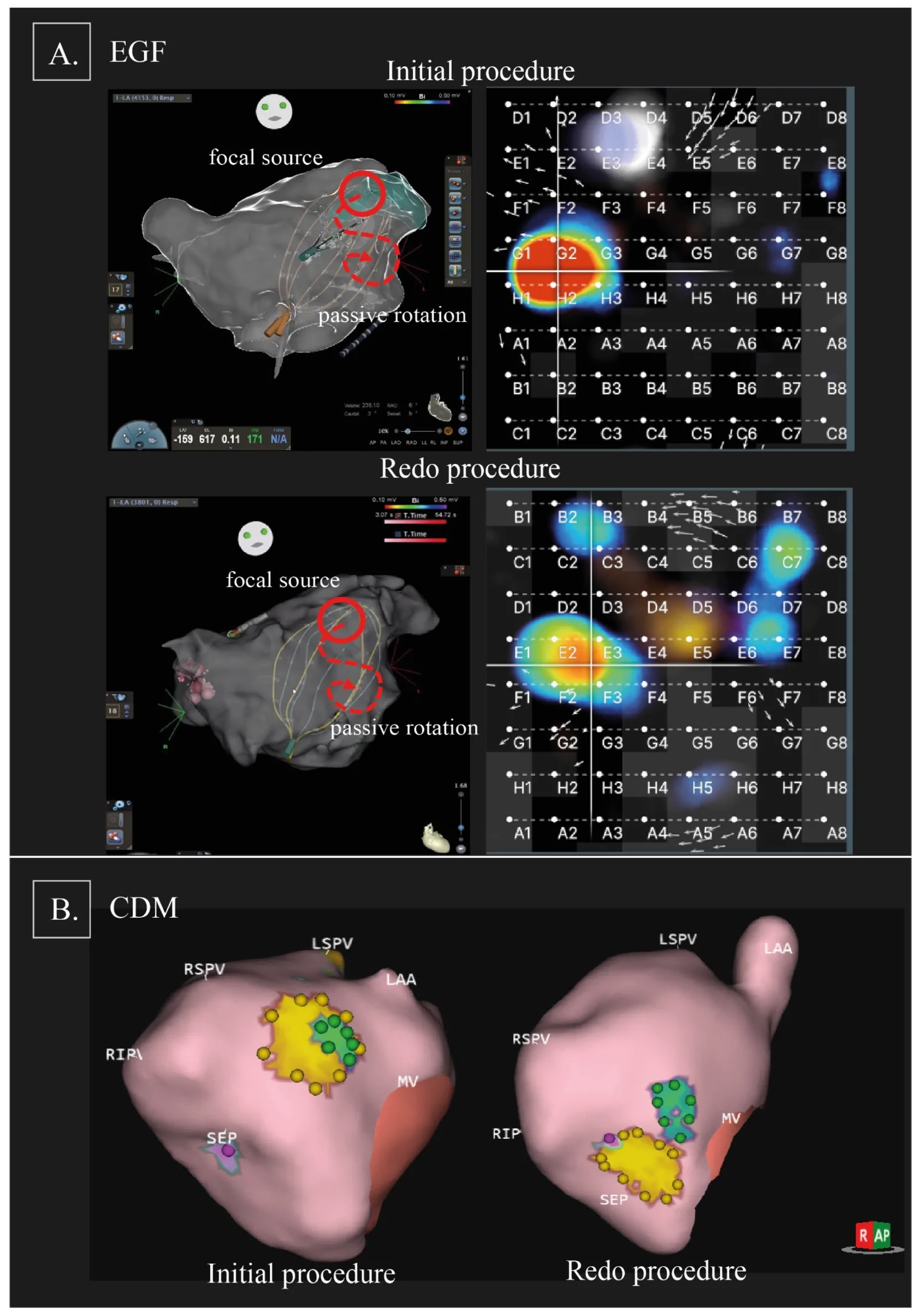
Examples of EGF and DCM maps. In panel (**A**), EGF maps are shown that were acquired during the initial and the redo procedures. Summary maps show an active extra-PV source around the G and E electrodes, which, on the left side of the panel, show approximately similar catheter positions and the same localization of the source. EGF mapping allows differentiation of active source and passive rotation, which is shown on the left side of the panel. In panel (**B**), CDM maps are shown acquired during the initial and the redo procedure. Sources marked with yellow and green colors were found in the same location between the RSPV and MV.

Regarding sources mapped with PAM systems, in 4 patients (44.4%), localization mapped at the repeat procedure corresponded with the localization of sources mapped during the index procedure (Figure 1). In two patients, no sources were identified during the second procedure. In the remaining 3 patients, the localization of sources was detected at different locations.

## 3. Discussion

The main finding of this case series is that persistent AF initiating and maintaining sources show spatiotemporal stability in approximately half of the cases; however, novel sources were identified during repeat procedures, suggesting possible disease progression.

In the current literature, success rates reported after CA for persistent AF are inconsistent. Even with the use of novel approaches, the long-term success rates of PVI-only procedures for patients with persistent AF remain suboptimal, and for PVI plus empiric adjunctive ablation, the outcomes are even more disappointing. Increasing evidence supports the existence of spatially localized extra-PV sources, suggesting that the optimal ablation strategy might be to detect and eliminate these AF initiating sources. It is also an important part of the strategy to understand the individual patient’s underlying atrial substrate that maintains AF. Because persistent AF is a complex arrhythmia that may be the end result of a broad range of pathophysiological processes, identifying and localizing extra-PV sources is a great challenge for electrophysiologists [7]. Detailed characterization of these sources remains sparse due to the limitations of the currently used mapping technologies [8,9,10]. In the last decade, technologies using global chamber mapping have been introduced, aiming to overcome some of the existing limitations of AF mapping [11,12,13].

The propagation history maps acquired by the CDM system identify and locate the discrete and coupled mechanisms responsible for the initiation of AF [11,14]. The recently published RECOVER AF study was designed to prospectively evaluate the safety and effectiveness of CDM in CA for redo procedures in patients with AF. The authors found that CDM reliably identified ablation targets, and 91% of patients who had only received a PVI ablation prior to treatment of extra-PV targets with the CDM system were AF-free at 12 months [6]. In a study published by Pope et al., results show that regular activation patterns identified by CDM are spatially stable and rotational activations are transient with low spatial stability. They concluded that stable regions of irregular activation may reflect underlying atrial structural abnormalities and might represent important sites for CA approaches [15].

The EGF mapping is the first technology to discriminate active and passive rotational electrical activity during endocardial mapping. As such, this technology may eliminate the unnecessary ablation of passive structures that do not contribute to AF initiation and maintenance and may improve post-ablation outcomes by identifying active AF drivers and/or triggers that may then be targeted for ablation. The recently published FLOW-AF trial is the first prospective, multicenter, randomized clinical study that evaluates the ability of the EGF mapping system to identify extra-PV sources in patients with persistent or long-standing AF who have failed prior PVI [16]. The most recently published clinical data suggest that patients treated with EGF-guided CA developed fewer AF recurrences, and EGF might offer a more targeted, patient-specific ablation strategy beyond PVI than adjunctive empiric lines and substrate ablation [17]. Spatiotemporal stability of an extra-PV source mapped with EGF mapping was demonstrated by our group previously in a patient during two procedures performed 18 months apart [18].

The CardioInsight mapping system noninvasively collects signals of electrical activation from the surface of the body and successfully localizes AF sources [19]. Because atrial signals are of lower amplitude compared to ventricular signals, in AF mapping, cumulative maps are used to locate the areas of focal breakthrough and reentrant drivers. In a study conducted by Osorio-Jaramillo et al., results show that driver location and activation patterns mapped with the CardioInsight system change constantly, and the authors found topographical changes in both focal and rotor activities [20].

In our study, the above-mentioned mapping systems were selected for their ability to provide global, chamber-wide visualization of atrial activation, which is crucial for identifying transient but spatially stable AF sources, particularly those located outside the pulmonary veins. The panoramic nature of these systems enables real-time mapping of the entire atrial surface, offering a more comprehensive view of complex activation patterns than is achievable with sequential, point-by-point techniques. In contrast, the widely used HD grid catheters offer localized, contact-based mapping [21]. While such catheters are effective and easier to implement in routine clinical practice due to their compatibility with existing electrophysiology lab infrastructure and workflows, they inherently provide only regional data. This is a significant limitation when trying to track spatiotemporal patterns or compare source locations across two procedures separated by time. Since CDM and CardioInsight use specific catheters, alternatives cannot be implemented. However, EGF is a catheter-independent software and can be used for the identification of active sources [22].

One important aspect of the present study is that the stability of extra-PV AF sources was observed using different mapping systems in some cases. However, the three mapping systems used in this study—CardioInsight, electrographic flow (EGF) mapping, and charge density mapping (CDM)—are based on different electrophysiological principles and signal-processing methodologies. Consequently, complete one-to-one concordance between systems cannot be assumed, and differences in sensitivity and specificity may influence source localization. Nevertheless, recurrent sources were frequently identified within similar broad anatomical regions, suggesting that at least some substrate characteristics may persist independent of mapping modality.

An additional observation of the present study was that right atrial (RA) sources were more frequently identified during index procedures than during repeat procedures. One possible explanation is that RA sources may represent more functional and transient electrophysiological phenomena rather than fixed substrate-driven drivers, making them less likely to persist over time. In contrast, dominant left atrial sources may be associated with more stable structural remodeling and fibrosis, potentially explaining their greater longitudinal reproducibility. Previous studies have demonstrated that right atrial (RA) rotational activity is frequently encountered in patients with persistent atrial fibrillation (AF) and may contribute to arrhythmia maintenance. Kis et al. reported a higher prevalence of RA than left atrial (LA) rotational activity (71% vs. 47%) and observed that AF termination following rotor-guided ablation was significantly associated with right-sided rotational activity [23]. These findings suggest that, in at least a subset of patients, the RA may serve as an important source of AF-maintaining activity rather than merely representing passive activation secondary to left atrial drivers.

## 4. Limitations

This study has several important limitations. First, the study population was small and consisted of only nine patients, limiting statistical power and generalizability. However, patients undergoing two consecutive electrophysiological procedures utilizing global atrial chamber mapping (GACM) are relatively uncommon, making this a rare and difficult-to-assemble cohort. Therefore, the findings should be considered exploratory and hypothesis-generating. Second, three different mapping systems were used (CardioInsight, EGF, and CDM), each relying on distinct acquisition methods and computational algorithms. These methodological differences may introduce variability in source detection and localization and could contribute to detection bias. Furthermore, because the retrospective nature of the study and differences in mapping platforms prevented precise mesh-to-mesh spatial co-registration between procedures, a fixed millimeter-based spatial tolerance threshold could not be reliably applied across all cases. Consequently, source stability was assessed based on concordant anatomical localization within predefined atrial regions rather than quantitative point-by-point spatial matching. Third, the study design cannot definitively distinguish whether stable sources identified during redo procedures represent incompletely ablated targets or intrinsically resilient arrhythmogenic substrate [18].

As noted in prior literature, the quality of electrogram recordings in contact-based mapping systems can be affected by the degree of catheter contact [24]. In particular, poor or inconsistent contact may lead to attenuated signal amplitudes and distorted substrate characterization, potentially introducing bias. Conversely, non-contact mapping modalities, such as CDM, are less susceptible to contact-related variability due to their design. Future advancements in real-time contact assessment and integration with electrogram analysis may further reduce this bias and enhance the fidelity of contact-based mapping approaches.

## 5. Conclusions

This is the first series of patients in which clear signs of spatiotemporal stability of AF sources are presented. The development of novel sources is also shown during the repeated procedure, suggesting disease progression in a significant number of patients.

## Figures and Tables

**Table 1:** Procedural properties.

	Gender	Age Group	Index Procedure				Time Between Procedures (Months)	Repeat Procedure			
			Year	Mapping System	Target	Additional Lines		Year	Mapping System	Target	Additional Lines
Patient 1	M	55–60	2019	CDM	LA: anterior wall (rotational activity)	-	40	2022	CDM	LA: anterior septum, posterior under LIPV	-
Patient 2	F	60–65	2018	CDM	LA: Posterior wall	Roof line, inferior line	12	2019	EGF	LA: Posterior wall	LA: anterior line RA: SVC isolation intercaval line
Patient 3	F	70–75	2020	EGF	LA: no source RA: low anterior	Carina line	4	2020	CDM	LA: anterior septum, posterior under RIPV, above CS RA: high IAS	-
Patient 4	M	40–45	2018	CardioInsight	LA: posterior wall, inferior to RIPV (rotational) RA: anterolateral (rotational)	LA: Roof line, infero-posterior line, BOX lesion RA: intercaval	4	2019	CDM	LA: Irregular and rotational posterior and anterior septal	Mitral isthmus line
Patient 5	M	55–60	2018	EGF	LA: Inferolateral and posterior parts LAA basis, posterior to the mitral isthmus, RA: (additional applications?)	-	38	2022	EGF	LA: under LIPV	Roof line, BOX lesion, Vein of Marshall alcohol ablation
Patient 6	F	65–70	2019	EGF	LA: superior transition to the LAA (rotational activity)	CTI line	19	2021	EGF	LA: rotational, anterior to LAA	-
Patient 7	M	70–75	2019	EGF	LA: lateral mitral annulus under the LAA RA: posterior to the IVC	LA: anterior line, BOX lesion RA: bicaval line	20	2021	EGF	LA: no source, RA: no source	defragmentation
Patient 8	F	55–60	2020	EGF	LA: posterior, LAA ridge, and LCPV RA: no source	LA: from WACA to mitral valve	25	2023	CDM	LA: anterior wall (focal, rotational slow activation), LAA ridge	-
Patient 9	M	70–75	2018	EGF	LA: posterior wall RA: lateral to the SVC	-	48	2022	CDM	LA: posterior wall	-

## Data Availability

The data that support the findings of this study are available from the corresponding author (T. Sz-T.) upon reasonable request.
